# Anti‐epilepsy drug therapy in a dox‐regulatable TDP‐43 mouse model of ALS‐FTD with seizures

**DOI:** 10.1002/alz.095761

**Published:** 2025-01-09

**Authors:** Irene Ra, Elizabeth Jia, Jaskreet Gujral, William Rodemer, Sílvia Porta, Virginia M.‐Y. Lee

**Affiliations:** ^1^ Center for Neurodegenerative Disease Research, Department of Pathology and Laboratory Medicine, University of Pennsylvania, Perelman School of Medicine, Philadelphia, PA USA

## Abstract

**Background:**

Cytoplasmic inclusions of TDP‐43 are the primary pathology in the majority of ALS and FTLD cases. Recent reports in cell and animal models suggest TDP‐43 pathology may enhance neuronal excitability, which could contribute to neurodegeneration via excitotoxicity. Dox‐regulatable rNLS8 mice express human TDP‐43 with mutations in the nuclear localization signal (hTDP‐43NLSm) to promote cytoplasmic accumulation. Preliminary electrophysiological data indicate these mice develop robust hyperexcitability in brain circuits along with generalized seizures. To test whether reducing hyperexcitability may be therapeutic, we administered the anti‐epilepsy drug, levetiracetam, to rNLS8 mice and assessed its effects on behavior and neurodegeneration.

**Method:**

hTDP‐43NLSm expression was induced by replacing doxycycline‐chow with normal mouse feed (off‐DOX) in a cohort of 16 adult rNLS8 mice, approximately equal numbers male and female. Starting 1 week off‐DOX, 100mg/kg levetiracetam (LEV) or 0.5% w/w hydroxypropyl methylcellulose with 1% Tween‐80 vehicle (VEH) was administered by daily gavage. At 4 weeks off‐DOX, mice were subjected to a behavior battery including cognitive (Y maze, elevated zero maze, 3‐chamber social interaction test) and motor (accl. rotarod, open field) tests. Brains were collected for histology at 6 weeks off‐DOX.

**Result:**

No improvement in behavioral deficits was observed in LEV treated mice. Preliminary analysis indicated a potential mild reduction in gross neurodegeneration. Mean hippocampus mass at 6 weeks off‐DOX was similar for both groups (21.7mg LEV, 22.mg VEH; Student’s t‐test, p = 0.84) but the LEV treated group showed a trend towards reduced cortical atrophy (mean motor cortex thickness on H&E stained sections: 943µm LEV, 870µm VEH; Student’s t‐test, p = 0.12).

**Conclusion:**

Daily levetiracetam administration was not effective in significantly reducing disease severity. Future studies will examine the effect of orthogonal approaches, such as AAV encoded chemogenetics, and characterize downstream mediators of TDP‐43 ‐induced hyperexcitability, which may serve as more potent and specific therapeutic targets.

